# The role of tryptophan metabolism and tolerogenic dendritic cells in maintaining immune tolerance: Insights into celiac disease pathogenesis

**DOI:** 10.1002/iid3.1354

**Published:** 2024-08-16

**Authors:** Fatemeh Asgari, Mahdi Khodadoust, Abdolrahim Nikzamir, Somayeh Jahani‐Sherafat, Mostafa Rezaei Tavirani, Mohammad Rostami‐Nejad

**Affiliations:** ^1^ Student Research Committee, Department of Clinical Biochemistry, School of Medicine Shahid Beheshti University of Medical Sciences Tehran Iran; ^2^ Gastroenterology and Liver Diseases Research Center, Research Institute for Gastroenterology and Liver Diseases Shahid Beheshti University of Medical Sciences Tehran Iran; ^3^ Department of Parasitology and Mycology, School of Medicine Shiraz University of Medical Sciences Shiraz Iran; ^4^ Laser Application in Medical Sciences Research Center Shahid Beheshti University of Medical Sciences Tehran Iran; ^5^ Proteomics Research Center Shahid Beheshti University of Medical Sciences Tehran Iran; ^6^ Celiac Disease and Gluten Related Disorders Research Center, Research Institute for Gastroenterology and Liver Diseases Shahid Beheshti University of Medical Sciences Tehran Iran

**Keywords:** celiac disease, glutens, tolerogenic dendritic cells, tryptophan metabolite

## Abstract

**Background:**

In mammals, amino acid metabolism has evolved to control immune responses. Tryptophan (Trp) is the rarest essential amino acid found in food and its metabolism has evolved to be a primary regulatory node in the control of immune responses. Celiac disease (CeD) is a developed immunological condition caused by gluten intolerance and is linked to chronic small intestine enteropathy in genetically predisposed individuals. Dendritic cells (DCs), serving as the bridge between innate and adaptive immunities, can influence immunological responses in CeD through phenotypic alterations.

**Objective:**

This review aims to highlight the connection between Trp metabolism and tolerogenic DCs, and the significance of this interaction in the pathogenesis of CeD.

**Results:**

It is been recognized that various DC subtypes contribute to the pathogenesis of CeD. Tolerogenic DCs, in particular, are instrumental in inducing immune tolerance, leading to T‐reg differentiation that helps maintain intestinal immune tolerance against inflammatory responses in CeD patients and those with other autoimmune disorders. T‐regs, a subset of T‐cells, play a crucial role in maintaining intestinal immunological homeostasis by regulating the activities of other immune cells. Notably, Trp metabolism, essential for T‐reg function, facilitates T‐reg differentiation through microbiota‐mediated degradation and the kynurenine pathway.

**Conclusion:**

Therefore, alterations in Trp metabolism could potentially influence the immune response in CeD, affecting both the development of the disease and the persistence of symptoms despite adherence to a gluten‐free diet.

## Graphical abstract

Tryptophan, one of the eight essential amino acids, has evolved as a primary regulatory node in controlling immune responses. Celiac disease (CeD) is a developed immunological condition caused by gluten intolerance. Tolerogenic dendritic cells emerge as pivotal conductors, inducing immune tolerance and facilitating T‐reg differentiation to safeguard intestinal immune tolerance against inflammatory responses in CeD and other autoimmune disorders.

## 1 INTRODUCTION

**Figure 1:**
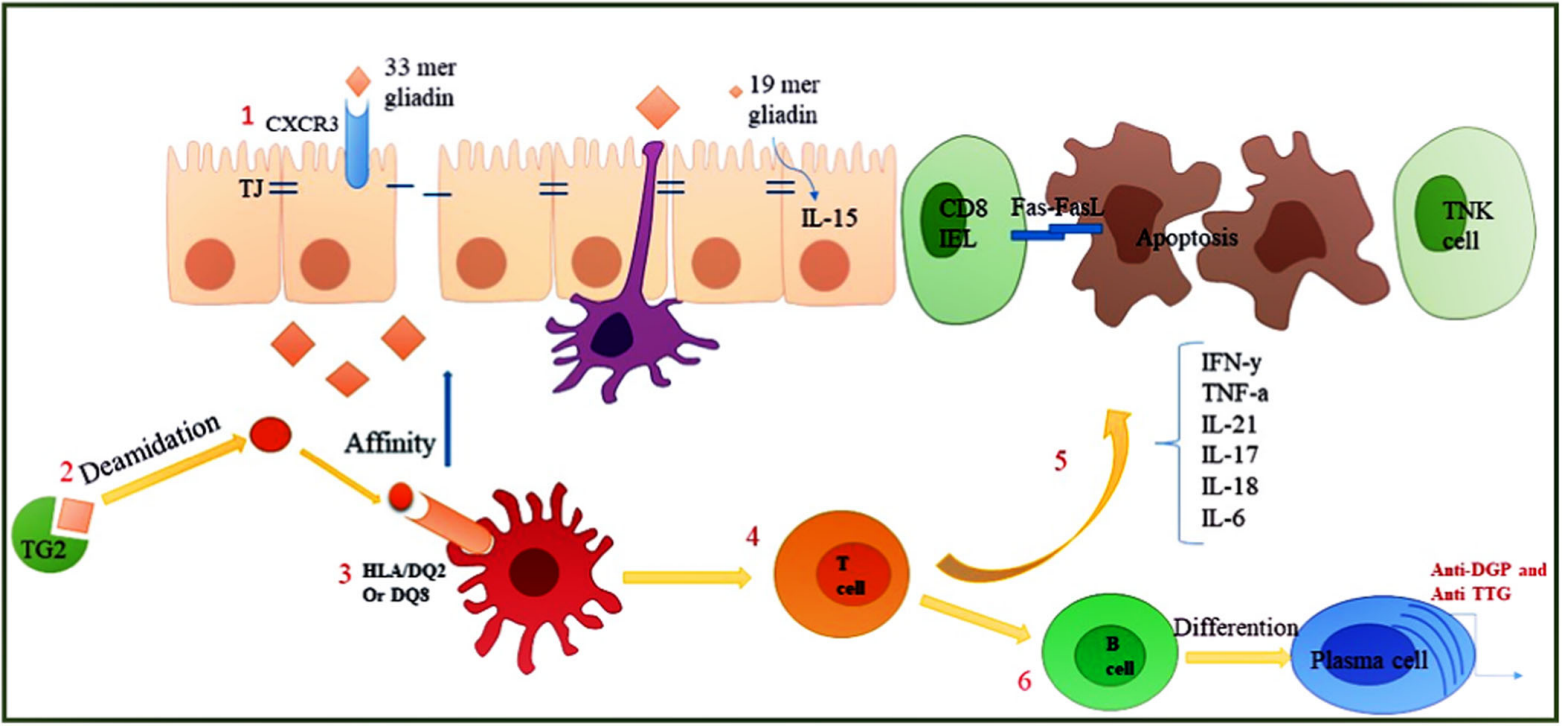
Pathophysiology of celiac disease. 1—Undigested Gliadin peptides move through paracellular and transcellular pathways and enter the intestinal submucosa. 2—In the lamina propria, tissue transglutaminase (tTG) deamidates Gliadin peptide. 3—Antigen‐presenting cells (APCs), which subsequently recognize deamidated Gliadin by their HLA‐DQ2 or HLA‐DQ8 molecules, inducing an immunological response. 4—As a result, Th1 and Th2 inflammatory pathways are activated. 5—CD8 and NK cells are stimulated by Th1 cells, and use the Fas/FasL system to induce apoptosis in the enterocytes. 6—B cells are stimulated by Th2 cells to develop into antibody‐producing plasma cells (anti‐tTG and anti‐Gliadin). Extracellular tTG and anti‐tTG interaction can result in further damage to the epithelium tissue.

Celiac disease (CeD), is defined as an immunity‐related chronic enteropathy of small intestine, being associated with dendritic cell (DC) accumulation, due to the dietary gluten protein intolerance which primarily affects the gastrointestinal tract in genetically vulnerable patients with HLA‐DQ2 or HLA‐DQ8 haplotypes.^1^ The soluble Gliadin and insoluble Glutenin are the two principal components of Gluten, which is a protein existing in rye, wheat, and barley. Both components have high Glutamine and proline contents.^2^, ^3^ CeD is linked to a variety of associated conditions, including osteoporosis, infertility, bloating, diarrhea, weight loss, anemia, depression, skin problems, and neurological diseases.^4^ CeD as a global disease is prevalence in the western countries with the approximation around 1%.^5^ The main causes of CeD pathogenesis include gluten protein intake, environmental conditions, mucus‐related immunities (of both innate and adaptive types), microbiota, and a special genetic component in the HLA‐DQ region on chromosome 6p21.3.^6^, ^7^, ^8^ The diagnosis of CeD typically involves a combination of clinical evaluation, serological testing, and histological examination of duodenal biopsies. The American College of Gastroenterology recommends the use of upper gastrointestinal endoscopy with multiple duodenal biopsies to confirm the presence of the disease in both children and adults. Available serological tests for CeD screening include anti‐tissue transglutaminase, anti‐endomysial, and anti‐deamidated gliadin peptide antibodies, with immunoglobulin A tissue transglutaminase generally considered to be the most sensitive and efficient test for CeD diagnosis. Additionally, an evaluation of genetic risk factors, including family history and the presence of HLA‐DQ2/DQ8, is also considered.^9^, ^10^ At present, the only effective treatment for CeD is complete gluten elimination from the diet. Adherence to a strict gluten‐free diet (GFD) can lead to symptom relief, serological remission, and histological recovery in most patients. However, a GFD may not always yield clinical or histological improvement, and maintaining a strict GFD can be challenging for some patients due to various reasons. Research focuses on developing pharmacological treatments to complement the diet and mitigate chronic gluten exposure.^11^ Despite ongoing research, there are still no FDA‐approved drugs specifically for CeD treatment. The complex process leading to CeD is being extensively studied and understood more clearly, opening up numerous potential targets for future treatments. Current research areas include the use of probiotics, peptide digestion, gluten sensitization, modulation of tight junctions, deamidation, and immune targeting.^12^, ^13^ In CeD, the incompletely digested peptides of Gluten can pass the epithelium layer of intestine through the transcellular or paracellular routes, in lamina propria. After possible deamidation by tissue transglutaminase (tTG), the potential of these peptides for binding the HLA‐DQ2/8 components of the antigen‐presenting cells (APCs) as well as Th1, Th2, and Th17 cells is enhanced, resulting in the secretion of pro‐inflammatory cytokines such as interleukin (IL)‐21 and interferon‐gamma (IFN‐γ), formation of various CeD antibodies as well as triggering the signals required for initiation of the acquired immunological reaction (Figure 1).^14^, ^15^ While the innate immunity is involved in the epithelial gut early defense, the acquired immunity in the intestine lamina propria triggers a cascade of immunological responses from macrophages, DCs, and B cells. Various subtypes of DCs are influential in CeD, such as plasmacytoid dendritic cells (pDCs) and tolerogenic DCs (tolDCs).^16^ TolDCs are central and peripheral tolerance agents in the body; they regulate homeostasis and inhibit excessive inflammation.^17^ In addition to their reduced stimulation capacity, tol DCs produce an impressive extent of anti‐inflammatory cytokines (i.e., IL‐10). They activate Foxp3+ T‐regs, which suppress T cells effector and reduce IFN‐γ, IL‐12, and TNF‐α production through their immunomodulatory properties.^18^ It has been suggested that tryptophan (Trp) catabolism is a key tolerogenic signal in T‐reg function, and its modulation through the expression of CTLA‐4 is thought to be crucial. It is important to note that the cytosolic heme protein, indoleamine‐2,3‐dioxygenase (IDO), which is the rate‐limiting enzyme in the catabolism of Trp, plays a pivotal role. It is instrumental in producing TGF‐β by DCs, facilitating T‐reg differentiation, and inducing tolerance in them. The aim of this study is to delve deeper into the complex interplay between CeD, DCs, and Trp metabolism. Specifically, the study seeks to understand how Trp catabolism, mediated by the enzyme IDO, influences the function of tolerogenic DCs and T‐regulatory cells in the context of CeD. By exploring these relationships, the study hopes to illuminate potential therapeutic strategies for CeD.

## 2 METHODS

### 2.1 Search strategy

We conducted a review of DCs, and Trp metabolism studies on CeD and searched the electronic databases of PubMed, EMBASE, and Scopus, relevant studies published up to April 2024, with the following search term: (“dendritic cell” OR “dendritic cells” OR “DC” OR “tolerogenic dendritic cells” OR “tolDC”), AND (“tryptophan” OR “Trp” OR “tryptophan metabolism”), AND (“celiac” OR “coeliac” OR “celiac disease” OR “CD” OR “CeD” OR “gluten enteropathy” OR “Gluten‐Sensitive Enteropathy” OR “Nontropical Sprue” OR “Celiac Sprue”). Additional articles were found by conducting a search through the reference lists of the studies that were included. Once the articles were gathered, further identification was done by applying the criteria for inclusion and exclusion. Inclusion criteria were set to prioritize English‐language studies that directly addressed the relationship between DC, Trp, and CeD. On the other hand, exclusion criteria were applied to filter out studies focusing on unrelated topics or lacking full text.

## 3 DENDRITIC CELLS DISTRIBUTION

The term “dendritic cell” was introduced by Steinman et al. in 1973. Until that time, it was thought that the cells comprising the innate and adaptive parts of the immune system were different entities.^19^ Since then, the understanding of DCs has grown over time. DCs, like other immune system cells, are divided into several categories or subsets that differ in origin, function, or both.^20^ The specific phenotypes of DCs are different and it basically depends on the architecture and location of the tissue in both mice and humans. Therefore, four primary DC subsets including conventional DCs (cDCs), monocyte‐derived DCs (moDCs), Langerhans cells (LCs), and plasmacytoid DCs (pDCs) exists.^21^ There is a common progenitor cell for all these subpopulations, called the monocyte, macrophage, and DC precursor (MDP), and Lymphoid organs, intestinal tract, skin, and blood contains different types of DCs.^22^ Intestinal DCs are found throughout the small and large intestine, in addition to mesenteric lymph nodes (MLNs), lymphoid follicles, and Peyer patches.^23^

### 3.1 Dendritic cells in celiac disease

DCs are the primary professional APCs that have a critical role in CeD and controlling immune reactions.^24^ Despite growing evidence of their immune activation and tolerance role, the DCs' function in CeD is baffling.^25^ Recent advancements in the pathogenesis of CeD have focused on the mechanisms through which tissue transglutaminase (the main autoantigen in CeD) deamidates certain glutamine residues of gliadin peptides, converting them into glutamic acid. This conversion increases their immunogenicity in the intestinal mucosa of CeD patients. This deamidation process is believed to be a key factor in the development of CeD. This modification imparts a negative charge to the gliadin, allowing it to bind more strongly to HLA‐DQ2 or HLA‐DQ8 molecules on APCs. Consequently, the neopeptides are exposed for recognition by T cells.^26^ The latter triggers a Th1 response leading to villous atrophy during activation. DCs perform two major functions in CeD. First, they present gliadin peptides as APCs to mucosal CD4^+^ T cells. The second possibility is that they may prolong the inflammatory response by interacting with lamina propria (LP) T cells.^25^ Small counts of CD103^+^ regulatory DCs and also plasmacytoid DCs (pDCs), as well as low interferon (IFN)‐alpha expression, define the celiac mucosa. On the other hand, macrophages and active cDCs aggregate in the tissue under inflammatory conditions, consequently activating the Gluten‐specific T‐cells.^27^, ^28^

### 3.2 Role of dendritic cells in peripheral tolerance

Tolerogenic DCs are also hypothesized to play a role in peripheral tolerance by assisting in inducing and/or preserving peripheral T‐reg cells through several different pathways.^29^ Simple T‐reg promoting conditions involve presenting modest levels of a cognate antigen without expressing costimulatory molecules and pro‐inflammatory cytokines. Aside from that, tolerogenic DCs can release anti‐inflammatory compounds. T cells may respond directly to such signals and/or modify their environment, such as their metabolic state, to fine‐tune their differentiation. The ability of intestinal DCs to induce T‐reg is just one of their specialized functions.^30^ CD4^+^CD25^+^FOXP3^+^T regulatory cells play a critical role in achieving and maintaining tolerance to dietary proteins. It is thought that intestinal LP CD103^+^ DCs express IDO at higher levels than other DCs and release RA along with IDO, which is essential for driving T‐reg and suppressing Th1 responses in the intestine.^31^ The presence of the required amino acids has a major impact on T‐reg production, differentiation, and function.^32^ T‐regs are produced when certain necessary amino acids are depleted in the surrounding environment, as a matter of fact Low doses of Trp limits T‐cell proliferation while increasing T‐reg production via mechanistic target of rapamycin (mTOR)‐dependent processes. This review summarizes recent improvements in our understanding of Trp metabolism for peripheral tolerance.

## 4 IDO AND FUNCTIONS

IDO1, a heme protein first isolated from rabbit intestine in 1967, is a rate‐limiting enzyme that catalyzes the oxidative cleavage of the indole ring of the essential amino acid Trp through the kynurenine pathway (KP). This process produces immunoregulatory metabolites, collectively known as kynurenines.^33^ In humans, the INDO gene on chromosome 8p12 encodes IDO, which is a 407‐amino acid cytoplasmic protein. IDO plays a crucial role in the immune system. It is thought to be an immunosuppressive mechanism in DC stem cells that suppresses T‐cell responses, induces immunosuppression and tolerance, and acts as a negative regulator in the prevention of autoimmune diseases.^34^ IDO is expressed in a wide range of tissues such as the lung, intestine, brain, and placenta. The IDO protein is encoded by a tightly regulated gene that responds to inflammatory mediators such as IFN‐γ, tumor necrosis factor‐alpha (TNF‐α), and lipopolysaccharide (LPS). IDO is expressed in APCs and its immunoregulatory function is achieved by DCs.^35^ IDO suppresses the function of CD4^+^ T cells by inhibiting cell proliferation, inducing apoptosis, and promoting differentiation into regulatory T lymphocytes. It also reduces the function of DCs by altering the function of APCs, regulating the expression of suppressive ligands (B7‐H1 ligand or CD95), and controlling the secretion of immunoregulatory cytokines (IL‐10 or TGF‐β).^36^

## 5 TRYPTOPHAN METABOLISM

The amino acid metabolism is crucial for preserving nitrogen in the body and maintaining physiologic amino acid levels, which cannot be stored when consumed in excess. The amino acid catabolic pathways have evolved to become vital immunity checkpoints throughout evolution.^37^ By using the novel function, self‐adaptive immune responses and exaggerated inflammatory responses can be controlled. A decrease in certain amino acids in the microenvironment, as well as the induction of biologically active metabolites, are required to achieve these immunoregulation effects. Rate‐limiting enzymes play a significant role in every degradative pathway, whose expression is normally tightly regulated. Recently, researchers have speculated that Trp catabolism by the kynurenine (Kyn) metabolic pathway is one of the mechanisms that mediate tolerance. The evidence suggests that the breakdown of Trp is essential for maintaining immune tolerance. To understand how Trp catabolism facilitates tolerance, two theories have been proposed. According to one theory, Trp breakdown reduces this amino acid's availability, inhibiting T‐cell proliferation. By depleting this essential amino acid, IDO hampers the biosynthesis of proteins and serotonin, limits microbial growth, and influences T‐cell proliferation. This could have potential implications for cell‐mediated immune responses, including the rejection of fetal allografts.^38^ Another theory holds that Trp catabolic products suppress immune cells by promoting apoptosis. Downstream metabolites of Trp catabolism, such as kynurenine, 3‐hydroxykynurenine, and 3‐hydroxyanthranilate, have been demonstrated to inhibit the proliferation of allogenic T cells, potentially through apoptotic mechanisms. Quinolinate, a catabolite of Trp, has been observed to trigger apoptosis in certain immune cells, including T‐helper type 1 cells, suggesting a role in diminishing T‐cell proliferation.^39^

### 5.1 Tryptophan origin and production

The l‐amino acid Trp, with a pyrrole‐type ring on its side chain and also a benzoic nucleus, was discovered by the English chemist F. Hopkins in 1901,^40^ as the biggest of the three aromatic amino acids.^41^ It is also classified as an amino acid of the ketogenic/glucogenic type according to its molecular formula, as C11H12N2O2.^41^ l‐Tryptophan is one of the eight essential amino acids which cannot be produced by human body and it should be obtained through diet to be utilized in protein synthesis.^42^ For human adults, the recommended daily dietary dose of Trp intake is in the range of 250–425 mg/day or 3.5–6 mg/kg (on average 4 mg/kg) of the body weight.^43^ Trp is usually found in bread, chocolate, cheese, oats, peanut, banana, milk, tuna, turkey, chicken, fish, and dried prunes.^42^

### 5.2 Intestinal tryptophan metabolism pathways

**Figure 2:**
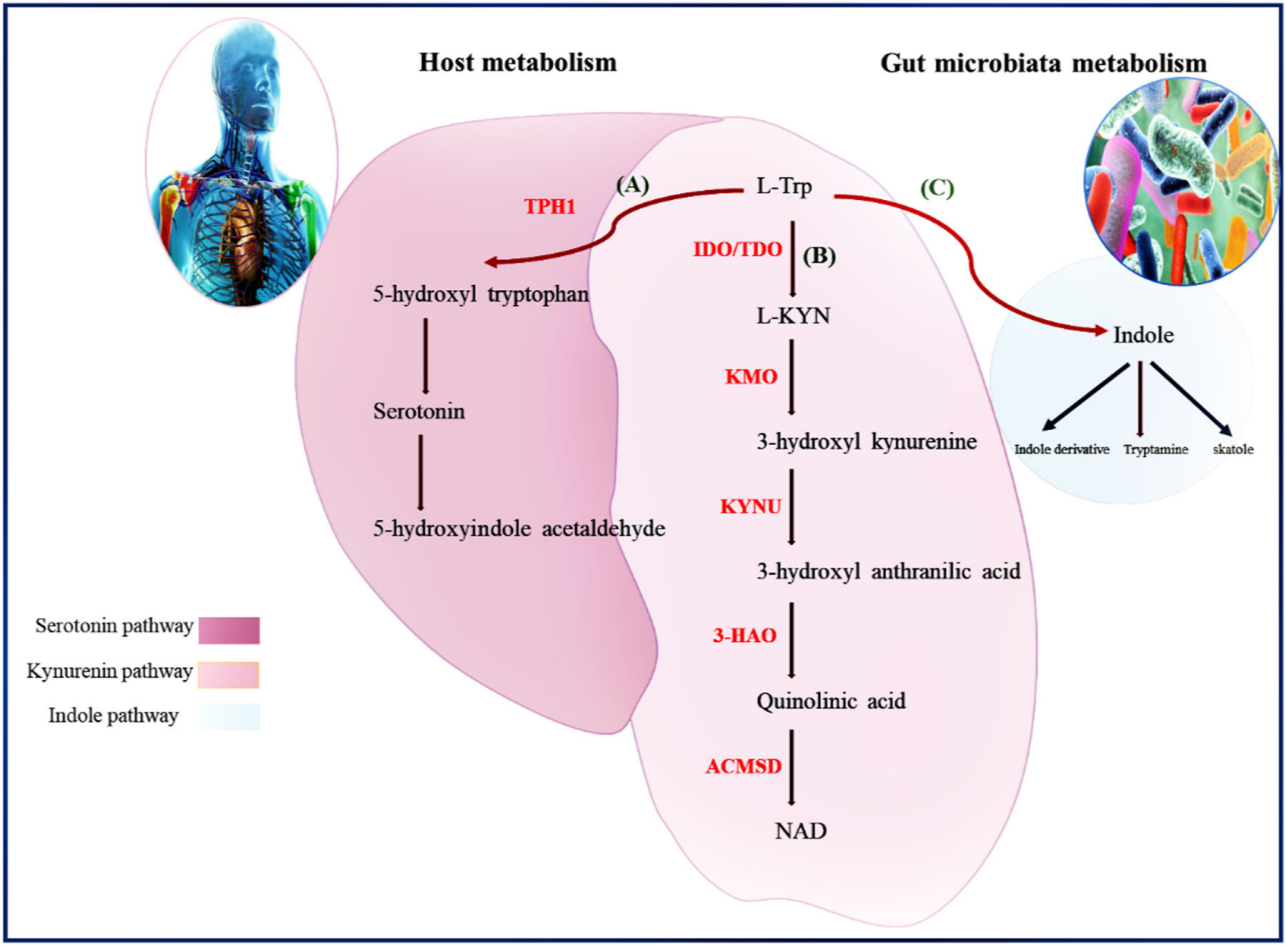
l‐Typthophan metabolism. The metabolic pathways of tryptophan involve the generation of bioactive metabolites through either the kynurenine pathway or the serotonin pathway in host organisms. Within gut microbes, tryptophan undergoes metabolic processes leading to the formation of indole and its derivatives. 3‐HAO, 3‐hydroxyanthranilate 3,4‐dioxygenase; ACMSD, alpha‐amino‐beta‐carboxymuconate‐epsilon‐semialdehyde decarboxylase; IDO, indoleamine 2,3‐dioxygenase; KMO, kynurenine monooxygenase; KYNU, kynureninase; TDO, tryptophan 2,3‐dioxygenase, TPH1, tryptophan hydroxylase.

Trp metabolism is mainly performed in the intestine. The digestive tract is covered by the host and also microbial cells that are involved in Trp ingestion and metabolization, generating key signaling molecules in the process (Figure 2). The gut microbiota (i.e., present in the gastrointestinal tract) metabolizes Trp into the aryl hydrocarbon receptor (AhR) ligands, while the host cells metabolize this molecule into kynurenine or 5‐hydroxytryptamine using indoleamine 2,3‐dioxygenase 1 (IDO1) or Trp hydroxylase 1 (TPH1), respectively. Each of these processes includes several metabolic intermediates that can perform signaling tasks on their own.^44^

#### 5.2.1 Indole pathway

The gut microbiota can convert Trp into indole derivatives, which are AhR ligands. AhR is a nuclear transcription factor that is activated by ligands and is presented throughout the body, but it is kept inactive in the cytoplasm. Following translocation to the nucleus after ligand binding, AhR affects the expression of some genes such as IL‐17, IL‐22, and Cyp1a1.^45^ According to a recent study, the expression of AhR in the gut mucosa is reduced under active CeD conditions. Nevertheless, the correlation between the gut microbiota and CeD, as well as the mechanisms of AhR regulation, has remained unclear.^46^

#### 5.2.2 Serotonin pathway

In mammalians, about 95% of the total 5‐hydroxy‐tryptamine, 5‐HT (also called a neurotransmitter serotonin) is presented in the gastrointestinal tract. Just 3% of the Trp obtained from diet is utilized in the 5‐HT synthesis process.^42^ It is known that 5‐HT is a biogenic factor that regulates important adaptive responses to environmental conditions and changes in the human CNS, including libido, impulsivity, cognition, aggressiveness, mood‐anxiety, nociception, body temperature, and feeding behavior. 5‐HT affects the activity of peripheral districts, including gastrointestinal function, inflammatory and immunological reactions, blood stem cell differentiation and also hemodynamic performance, in addition to its role as a neurotransmitter.^47^, ^48^ In the serotonin route, the aromatic Trp core is preserved^40^, ^49^ with the catalyzing role of TPH. It is known that TPH1 and TPH2 are active in the peripheral tissues of the body (such as skin and gastrointestinal tract), and in the neuronal cells, respectively.

#### 5.2.3 Kynurenine pathway

The KP metabolizes up to 90% of Trp after protein synthesis, and the related intermediates are AhR ligands, which mainly affect the immunological reactions.^41^ Activation of indolamine and tryptophan‐ 2,3‐dioxygenase (IDO and TDO, respectively) restricts kynurenine initiation. APCs such as B lymphocytes, macrophages, and DCs, as well as tumor cells, vascular endothelial cells, and epithelial cells are all induced by pro‐inflammatory cytokines to produce IDO, which activates the Trp catabolic Kyn pathway. The immunological response is modulated when Trp is depleted and Kyn is produced at the same time.^50^, ^51^

## 6 TOLERANCE MECHANISMS BY TRYPTOPHAN METABOLISM

**Figure 3:**
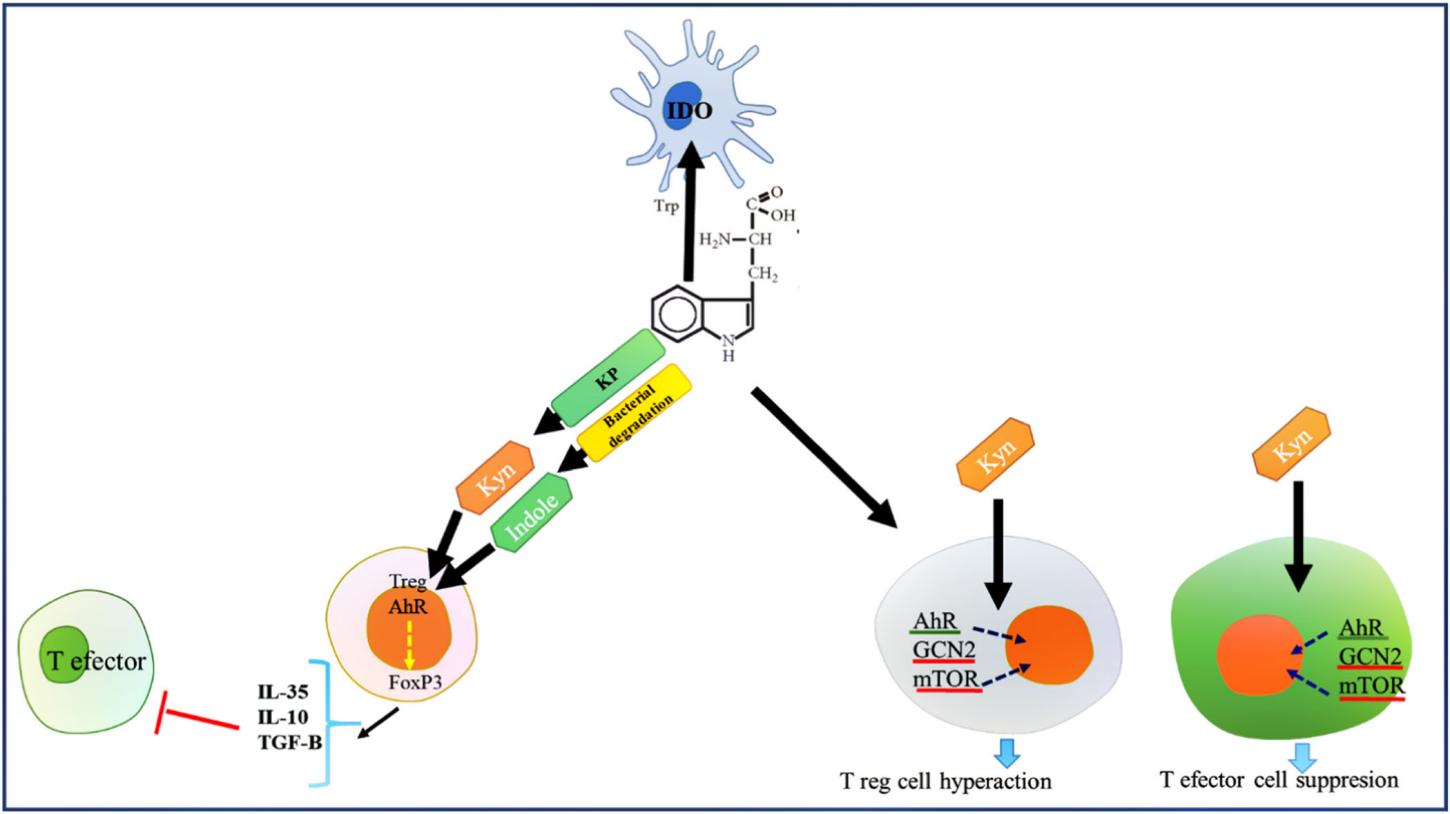
Trp metabolism and IDO. As a result of Trp metabolism, ligands of AhR are produced via KP and microbially induced breakdown that affects T‐reg development. The release of Kyn and consumption of Trp are caused by IDO activity in APCs. Immune suppression and tolerance are caused by the effects of such metabolic cues on T‐reg and effector T cells through the release of Kyn, and the consumption of Trp (via GCN2 and mTOR, as two amino acid sensors) as well. APC, antigen‐presenting cell; IDO, indoleamine 2,3‐dioxygenase; mTOR, mechanistic target of rapamycin.

Trp metabolism through metabolites regulates hyper inflammation and promotes immunological tolerance over time. These effects are contingent on IDO ability as a key inhibitor of the immune response, to prevent potentially exaggerated inflammatory reactions to danger signals.^52^ Signal transducer and activator of transcription‐1 (STAT1), interferon regulatory factor‐1 (IRF1), and nuclear factor‐kappa B (NF‐KB) are responsible for regulating IDO expression upstream.^53^ Kynurenines, catabolic products of IDO, inhibit immunity by modulating T effector cell (Teff) anergy and stimulating the proliferation of T regulatory cells. Tolerogenic pathways mediate immune responses under various stress conditions. In IDO‐capable cells, this balance has a direct impact on immunological and metabolic signaling routes, generating an anti‐inflammatory reaction. This enzyme has the ability to suppress T‐cell response and T‐reg formation, suppress the proliferation of effector T cells, induce their apoptosis, and ultimately induce immunological tolerance.^54^, ^55^ A synthetic Trp metabolite that promotes T‐reg activation has been successfully used to treat autoimmune neuroinflammation.^56^ Furthermore, establishing a local or systemic milieu with a high Kyn content and low Trp level alters the performance of surrounding cells, such as T cells.^57^ The role of IDO‐producing cells in preventing autoimmune disorders has been proposed.^58^ When an autoimmune disorder has developed, chronic inflammation can result in sustained IDO production, even when IDO is unable to control immune dysregulation. In previous studies, the following two mechanisms have been proposed for IDO‐related suppressive mechanisms, which are also illustrated in Figure 3.^59^

### 6.1 Downstream metabolites have a direct interaction with immunity‐related cells, via the aryl hydrocarbon receptor

#### 6.1.1 T‐reg differentiation through indole ligands

It has been shown that indole and its derivatives reach intestinal epithelial cells and enter the circulatory system, where immune cells recognize and activate the AhR signaling pathway. As demonstrated in Figure 2, AhR signaling induces the proliferation of CD4^+^CD25^+^Foxp3^+^ T‐regs, contributing to adaptive immune tolerance by inhibiting the function of activated T‐cells. Among the studies published by Lamas et al. in 2020, experiments conducted on nonobese diabetic (NOD) mice expressing the DQ8 CeD susceptibility gene (NOD/DQ8) demonstrated a significant modulation of intestinal microbiota with an enriched Trp diet. This modulation of gut microbiota resulted in higher AhR ligand production, thereby reducing immune pathology in NOD/DQ8 mice exposed to gluten.^60^

#### 6.1.2 T‐reg differentiation through kynurenine pathway

Not only Kyn, but also other metabolites, for instance quinolinic acid (QA), anthranilic acid (AA), xanthurenic acid (XA), Kynurenic Acid (KA), and 3‐hydroxykynurenine (3‐HK) are generated by the KP route of Trp catabolism. A number of KP metabolites are linked to AhR, inducing the expression of FoxP3 and promoting FoxP3+T‐reg production and differentiation. T‐reg activity can be also triggered by 3‐HK and its metabolites, such as pyridine‐2,3‐dicarboxylic acid. High Kyn level enhances the transition of naive CD4^+^ T‐cells into T‐regs, which is consistent with the long‐term synergistic effect of Trp deficiency.^36^ Francisco et al. discovered that the dioxin receptor AhR regulates T‐reg and Th‐17 differentiation according to its ligands.^61^

### 6.2 Depletion of Trp activates general control non‐depressible 2 stress kinase, while inhibiting the mechanistic target of rapamycin complex 1

Trp levels are important in the metabolic‐based modulation of inflammation, because they affect two major nutrient‐sensing systems. Depletion of Trp stimulates the general control non‐depressible 2 stress kinase (GCN2), while inhibiting the mTOR complex 1 (mTORC1).^62^ Depletion of Trp due to an imbalance or the lack of amino acids leads to the activation of GCN2, which phosphorylates eukaryotic initiation factor 2 (eIF2), as its downstream target. In APCs, after the mentioned process, some anti‐inflammatory cytokines (e.g., IL10) are produced and T‐regs are activated.^63^, ^64^ Natural killer cells and also T lymphocytes can be inhibited by kynurenine. Such signaling effect of GCN2/IDO is not limited to immune cells. It also plays a role in minimizing inflammatory tissue damage, for example, by activating autophagy.^65^, ^66^ Hence, Trp metabolism activation is associated with immunosuppressive and also anti‐inflammatory impacts that restore the homeostasis of the living organism. Trp depletion, inhibits mTORC1 (as a major regulatory agent in cellular processes), that is particularly important for proteosynthesis and also mass regulation of skeletal muscles. mTORC1/IDO signaling has also a critical role in the activation of immune cells.^51^, ^67^ In a study, Fallarino et al. demonstrated that Trp deprivation, combined with Trp catabolites, induces GCN2 kinase‐dependent downregulation of the TCR ζ‐chain in murine CD8^+^ T cells. This condition also induced a regulatory phenotype in naive CD4^+^CD25^−^ T cells through TGF‐β induction of Foxp3.^66^

## 7 MECHANICS OF T‐REG CELLS CONTROL THE IMMUNE RESPONSE IN CELIAC DISEASE

**Figure 4:**
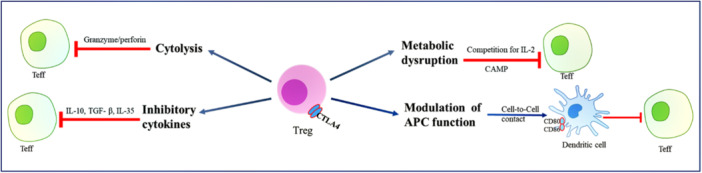
Mechanics of T‐reg cells control the immune response. T‐reg cells use multiple mechanisms, including (I) inhibition by immunoregulatory cytokines; (II) inhibition by cytolysis of effector cells; (III) inhibition by metabolic interruption; and (IV) modulation of dendritic cell function and maturation.

During inflammatory diseases, T‐reg cells are attracted to the site of inflammation and direct the immune response by suppressing Teff cells. To mediate their suppressive effects, T‐reg cells use multiple mechanisms (see Figure 4), including (I) inhibition by immunoregulatory cytokines; (II) inhibition of effector cells by cytolysis; (III) inhibition by metabolic interruption; and (IV) modulation of DC function and maturation. These mechanisms contribute to T‐regs maintaining intestinal homeostasis, autoimmune tolerance, and immune stability.^56^ The regulatory functions of T‐regs in CeD have been examined in many studies, but more research is needed to elucidate their other functions.

### 7.1 Inhibition by inhibitory cytokines

T‐reg cells release immunomodulatory cytokines, such as IL‐10, IL‐35, and TGF‐β. In addition to its role as an immunomodulatory cytokine, IL‐10 can be induced by catecholamines and act as a paracrine or autocrine signal.^68^ IL‐10 exhibits various biological activities, including immunosuppression, anti‐inflammation, and immunomodulation.^69^ It inhibits the expression of MHC I on B and T cells, as well as DCs, as a result, all of them contribute to inflammation.^70^

TGF‐β regulates the function of several types of immune cells. It has been proposed that it suppresses (i) the differentiation of effector T cells, (ii) the development of regulatory T cells or Th17 cells from naive T cells, (iii) the proliferation of T and B cells, and (iv) the activity of macrophages, DCs, and NK cells.^71^

IL‐35 belongs to the IL‐12 family and is a heterodimeric cytokine. It has two known functions: one is to suppress the proliferation of T helper cells, and the other is to promote the transformation of naive T cells into highly suppressive T‐reg cells.^72^ It was recently shown that this cytokine can also induce B lymphocytes to become regulatory B cells.

### 7.2 Inhibition by cytolysis

As a result of their production of granzyme B, T‐reg cells induce apoptosis in effector T cells. Granzyme B can also enter cells via endocytosis, activated by the mannose‐6‐phosphate receptor. Apoptosis is induced by granzyme B through caspase‐dependent or ‐independent mechanisms when it reaches a target cell.^73^

### 7.3 Inhibition by metabolic disruption

#### 7.3.1 Inhibition of proliferation by IL‐2 competition

T‐cell proliferation is primarily regulated by IL‐2. The IL‐2 receptor complex consists of three subunits (α, β, and γ). The alpha chain of this complex (IL‐2Rα) is required to increase IL‐2 affinity to its receptor. T‐reg proliferation, survival, and function are dependent on IL‐2. Due to their constitutive expression of the alpha chain of IL‐2 (CD25), T‐reg cells have a higher affinity for this cytokine. As T‐regs cannot produce IL‐2 and must source it exogenously, they compete with Teff cells for IL‐2. Consequently, T‐regs disrupt the proliferation of Teff cells, causing metabolic interruption and cell death.^74^

#### 7.3.2 cAMP‐mediated immunosuppression

Second messengers, such as cAMP, are essential for T‐reg suppression in the cell. T‐regs can increase cAMP levels in Teff cells through two major mechanisms. The first involves T‐regs transferring cAMP into Teff cells through gap junctions, while the second mechanism involves ATP being converted into adenosine, and this adenosine then attached to the receptors on the Teff cell surface, causing the level of intracellular cAMP to rise. Cellular proliferation and differentiation, as well as interferon gene expression and IL‐2 expression, are inhibited by cAMP in Teff cells.^75^

### 7.4 Inhibition by targeting dendritic cells

#### 7.4.1 Interaction through CTLA‐4

CTLA‐4 (cytotoxic T lymphocyte antigen‐4) is a receptor that T‐regs can use to interact with DCs. DCs express CD80 and CD86, which bind to CTLA‐4 with high affinity. For T cells to be fully activated, CD28 interacts with CD80 and/or CD86 on the APCs, which are transmembrane proteins of the immunoglobulin gene family. A disturbance in this interaction can result in T‐cell anergy or non‐responsiveness. As CTLA‐4 binds with higher affinity to CD80/CD86 ligands, it suppresses the activation of Teff cells. INF‐γ, a potent inducer of IDO, is produced during this interaction.^76^

#### 7.4.2 Interaction through LAG3

T‐regs express lymphocyte‐activation gene 3 (LAG3, CD223) on their cell surface. The binding interaction between LAG3 and MHC class II molecules prevents DC maturation and function.^77^ Additionally, the conjoint union of CTLA‐4 and LAG3 promotes a tolerogenic phenotype in DCs.

## 8 DENDRITIC CELLS AND AUTOIMMUNE RESPONSES: ONSET AND PREVENTION

DCs, a crucial component of the immune system, play a significant role in the mechanisms involving T‐reg cells. The activation or control of the immune response depends on the DC's phenotype as a result of its interactions with T‐reg cells. DCs acquire a tolerogenic phenotype that enhances T‐reg cell generation and establishes a suppressor microenvironment. The competition between T‐reg and effector cells for DC ligands provides an additional control mechanism for immune responses. TolDCs often express small levels of costimulatory CD80, CD86 molecules and high levels of co‐inhibitory molecules (programmed cell death ligand‐1 and ‐2 (PDL‐1 and PDL‐2, respectively), suppress active T cells and secrete more TGF‐β and also IL‐10 and, and less amounts of inflammatory cytokines including TNF‐α, IL‐1, IL‐12, and IL‐6. Aside from the activation of T‐reg cells and release of IL‐2, some enzymes (for instance, IDO1 and retinaldehyde dehydrogenase) play parts in the inhibition of Th1‐based autoimmunity.^78^, ^79^ All of these elements work together to preserve a tolerogenic DC phenotype when self‐antigens are presented. On rare occasions, the mentioned mechanisms, which prevent the detection of self‐antigens by the immune system in humans, may fail. This error can generally be caused by the presentation of self‐antigens in the context of danger‐type signals, breaking the tolerance. Although various mechanisms related to the DC start of autoimmunity have been proposed for each autoimmune illness, they are still unknown. For example, in the CeD, virus‐based infections and also gene‐related abnormalities of DC subtypes have been considered as autoimmunity initiators.^20^ According to previous studies, reovirus infection can disrupt the intestinal immune balance by suppressing the conversion of peripheral regulatory T cells and promoting Th1 immunity to gluten in food, which can lead to the development of CeD. In particular, some reovirus strains can prevent the development of tolerogenic T cells, which are essential for maintaining oral tolerance to food antigens such as gluten. In addition, higher levels of reovirus antibodies and also IFN regulatory factor 1 (IRF1) gene expression have been found in individuals with CeD, which is associated with the loss of oral gluten tolerance. This finding implies that CeD development can be significantly influenced by reovirus infections.^80^ Brigleb et al. have found that intestinal Strain type 1 Lang (T1L)‐infected mice attract and activate NK cells in MLNs in a type I IFN‐dependent manner. After infection, NK cells are activated and subsequently produce type II IFN, thereby supporting IFN‐stimulated gene expression in MLNs and triggering T and DC inflammatory responses.^81^ The role of gut microbes, including protists, in preventing virus‐induced loss of oral gluten tolerance has been recently investigated using a mouse model of the disease. It has been reported that the commensal intestinal protist Tritrichomonas arnoldi can prevent virus‐induced loss of oral tolerance by suppressing a pro‐inflammatory mechanism in CD103^+^ DCs, thereby preserving immune balance through inhibiting Th1 responses and enhancing regulatory T‐cell responses. This shows that certain gut microbes may be able to prevent the onset of CeD by reducing the impact of viral infections on gluten tolerance.^82^ Among all amino acids, only Trp and its metabolism have been related to autoimmunity. According to several studies, Trp catabolism takes place in the inflamed sites of tissues, where IDO expression can have an anti‐inflammatory effect by reducing tissue damage in these regions.^83^

## 9 TRYPTOPHAN METABOLITES INVOLVED IN INFLAMMATION

Inflammation and tissue damage are also controlled by Trp metabolites. Serotonin, for example, has been linked to intestinal inflammation.^84^ Two other Kyn‐related metabolites (namely cinnabarinic acid and 3‐hydroxyanthranilic acid) are linked to encephalomyelitis of autoimmune type and inflammation of vessels.^85^ NAD+ protects renal kidney damages and modulates macrophage‐mediated immune reactions, while indoles and Kyns are key players in the gastrointestinal and neurological inflammation.^86^ According to Table 1, both in vitro and in vivo studies have investigated the effects of Trp metabolites on inflammation and T‐reg differentiation.

**Table 1:** The effects of Trp metabolite on regulates hyper inflammation and T‐reg differentiation.

Study	Type of Trp metabolite	Model	Result	Reference
Zaher et al.	3Hydroxykynurenine	In vitro and murine	Reduces proliferation of CD4 T‐cells Promotes the development of T‐reg	87
Bracho‐Sanchez et al.	recombinant human IDO	In vitro	decrease in IL‐12p70 secretion Maintain IL‐10 secretion at a basal level	88
Salimi et al.	kynurenic acid	In vitro	Reduced of IL‐17 and IL‐23 production	89
Manni et al.	l‐kynurenine	In vitro	Increased TGF‐β production	90
Lee et al.	3‐hydroxyanthranilic acid	In vitro	Reduced levels of IL‐12, IL‐6, and TNF‐α A reduction in the maturation markers CD40, CD80, CD86, and I‐A	91
Fallarino et al.	kynurenines	In vitro and in vivo murine	CD4^+^CD25^+^ cells exhibit a regulatory phenotype	56
Islam et al.	0.5% tryptophan diet	In vivo Female wild‐type mice	Reduced the mRNA expression of IL‐6, TNF‐α, IL‐1β and the chemokines Ccl2, Cxcl1 and Cxcl2 Regulating Foxp3 and IL‐17 transcripts	92

## 10 TRYPTOPHAN METABOLISM IN CELIAC DISEASE

As a result of CeD, patients' plasma was found to have altered amino acid metabolism and its metabolites. This alteration is believed to be due to the decreased ability of gut microbes in CeD patients to metabolize dietary Trp.^93^, ^94^ The exact cause of this altered Trp metabolism is not fully understood, but it could be a combination of genetic predisposition and environmental factors, such as diet and gut microbiota.^95^, ^96^ The initiation of a GFD in CeD patients can lead to a reduction in Trp intake. This reduction could potentially have implications for CeD patients with altered Trp metabolism. Trp is a precursor to serotonin, a neurotransmitter that plays a key role in mood regulation. Therefore, a reduction in Trp intake could potentially affect mood and lead to depressive symptoms.^97^ Additionally, Trp is a precursor to the production of niacin (vitamin B3), which is essential for metabolism and the health of the skin, nervous system, and digestive system.^98^ Also, Trp and its metabolites play a key role in modulating inflammation and protecting the gut barrier. Therefore, a reduction in Trp intake could potentially exacerbate inflammation in CeD patients.^99^ Numerous research has shown that Trp metabolism is altered in CeD patients. According to Van Hees et al., a long‐term GFD significantly reduced vegetable protein intake in CeD patients, and levels of essential amino acids such as Trp were lower in these patients.^100^ In another study, Lamas et al. described that patients with active CeD produce lower levels of AhR ligands and their intestinal AhR pathway is less activated.^60^ A report has been published regarding the enhanced expression of IDO throughout the small intestine of individuals with CeD, which can be a key element in the mechanism that causes tolerance to dietary antigens. In fact, this increase may result from the persistent antigen stimulation used to control the inflammatory cause.^101^ However, it is important to note that these are potential implications and more research is needed to fully understand the impact of reduced Trp intake in CeD patients following a GFD.

## 11 CONCLUSION

Numerous studies have highlighted the role of certain amino acids and vitamins in modulating the immune response and various inflammation and immunity‐related pathological conditions in the gastrointestinal tract.^92^, ^102^, ^103^ Trp, along with its metabolites especially kynurenine, has been demonstrated to significantly contribute to immune regulation. Trp is metabolized through the KP, which becomes activated in response to inflammation and immune reactions. This pathway is regulated by the enzyme IDO, which is stimulated by pro‐inflammatory cytokines. In the context of CeD, an autoimmune disorder triggered by the ingestion of gluten in genetically predisposed individuals, the immune system plays a pivotal role. When an individual with CeD consumes gluten, their immune system reacts abnormally, resulting in damage to the small intestine. Trp metabolism can potentially influence this immune response in several ways. First, the conversion of Trp into kynurenine by IDO can result in local Trp depletion. This has been demonstrated to inhibit the proliferation of T cells, which are key contributors to the immune response. Second, certain metabolites of the KP have been shown to promote the development of Tregs, which play a role in maintaining immune tolerance. When a patient with CeD initiates a GFD and experiences a reduction in Trp intake, this could potentially exacerbate any existing deficiencies or imbalances in Trp metabolism. Therefore, alterations in Trp metabolism could potentially influence the immune response in CeD, affecting both the development of the disease and the persistence of symptoms despite adherence to a GFD. However, more research is needed to fully understand these mechanisms and translate them into effective treatment strategies for CeD patients.

## AUTHOR CONTRIBUTIONS

**Fatemeh Asgari**: Conceptualization; visualization; writing—original draft. **Mahdi Khodadoust**: Conceptualization; visualization; writing—original draft. **Abdolrahim Nikzamir**: Investigation; writing—review and editing. **Somayeh Jahani‐Sherafat**: Visualization; writing—review and editing. **Mostafa Rezaei Tavirani**: Writing—review and editing. **Mohammad Rostami‐Nejad**: Conceptualization; investigation; writing—review and editing.

## CONFLICT OF INTEREST STATEMENT

The authors declare no conflict of interest.
